# Partial upper sternotomy for extensive arch repair in older acute type A aortic dissection patients

**DOI:** 10.1186/s12872-022-02511-1

**Published:** 2022-03-21

**Authors:** Zhihuang Qiu, Jun Xiao, Qingsong Wu, Tianci Chai, Li Zhang, Yumei Li, Liangwan Chen

**Affiliations:** 1 Department of Cardiac Surgery, Union Hospital, Fujian Medical University, Fuzhou, 350001 Fujian China; 2 Key Laboratory of Cardio-Thoracic Surgery, Fujian Medical University, Fujian Province University, Fuzhou, 350001 Fujian China; 3 Fujian Provincial Special Reserve Talents Laboratory, Fuzhou, 350001 Fujian China; 4 Department of Physiology and Pathophysiology, School of Basic Medical Sciences, Fujian Medical University, Fuzhou, China; 5 Department of Toxicology, Fujian Center for Evaluation of New Drug, Fujian Medical University, Fuzhou, China

**Keywords:** Acute type A aortic dissection, Older patients, Full sternotomy, Partial upper sternotomy

## Abstract

**Objectives:**

The partial upper sternotomy (PUS) approach is acceptable for aortic valve replacement, and even aortic root operation. However, the efficiency of PUS for extensive arch repair of acute type A aortic dissection (AAAD) in older adult patients has not been well investigated.

**Methods:**

Between January 2014 and December 2019, 222 older adult patients (≥ 65 years) diagnosed with AAAD went through extensive arch repair, among which 127 received PUS, and 95 underwent full sternotomy (FS). Logistic regression analysis was used to identify risk factors for early death, and negative binomial regression analysis was applied to explore risk factors related to post-operative ventilator-supporting time and intensive care unit stay time.

**Results:**

Total early mortality was 8.1% (18/222 patients). The PUS group had shorter Cardiopulmonary bypass time (133.0 vs.155.0 min, *P* < 0.001), cross-clamp time (44.0 vs. 61.0 min, *P* < 0.001) and shorter selective cerebral perfusion time (11.0 vs. 21.0 min, *P* < 0.001) than the FS group. Left ventricle ejection fraction  < 50% (odds ratio [OR] 17.05; 95% confidence interval [CI] 1.87–155.63; *P* = 0.012) and malperfusion syndromes (OR 65.83; 95% CI 11.53–375.86; *P* < 0.001) were related to early death. In the multivariate model, the PUS approach contributed to shorter ventilator-supporting time (incidence rate ratio [IRR] 0.76; 95% CI 0.64–0.91; *P* = 0.003), when compared with the FS group.

**Conclusions:**

The early results of emergency extensive arch repair of AAAD via PUS in older adult patients were satisfactory. However, the long-term results remain to be investigated.

## Introduction

Formidable healthcare challenges brought about by aging are increasing worldwide, including western countries and China [1, 2]. Cardiac surgeons will be increasingly confronted with treating older adult patients who were suffering from cardiovascular diseases, especially acute type A aortic dissection (AAAD). The latest advances in surgical techniques, anesthesia, cardiopulmonary bypass techniques, and postoperative management had significantly reduced the operative mortality of AAAD during the last decade, however some studies continue to report advanced age as a risk factor for mortality of AAAD [3, 4]. The extensive arch repair of AAAD in older adults patients remains a challenge for cardiac surgeons.

Some highly experienced institutions have reported satisfactory results of surgical repair for older adult patients with AAAD. Since 2014, our group has applied a hemiarch replacement combined with a modified triple-branched stent graft for extensive arch repair for AAAD through a full sternotomy (FS), with excellent short-term results [5–7]. This novel procedure simplifies the extensive arch repair of the AAAD procedure, reducing the aortic cross-clamping time and hypothermic circulatory arrest time. The partial upper sternotomy (PUS) approach is acceptable for cardiac surgery as it reduces surgical trauma, while maintaining chest stability and improving the postoperative course. To this end, our institution has achieved extensive arch repair of AAAD through PUS in older adult patients since 2017. The aim of this study was to evaluate the efficiency of extensive arch repair of AAAD through PUS compared to FS in older adult patients.

## Patients and methods

This study was approved by the ethics committee of union hospital, Fujian Medical University, China. Between January 2014 and December 2019, 222 older adult patients (≥ 65 years) underwent hemiarch replacement combined with a modified triple-branched stent graft for extensive arch repair for AAAD. Of those, 127 patients received the PUS procedure, and 95 patients underwent the FS procedure.

Diagnosis of AAAD was made with contrast-enhanced computed tomography angiography (CTA) and echocardiography. Patients who met the following criteria would undergo a hemiarch replacement combined with a modified triple-branched stent graft for extensive arch repair both for PUS and FS: (1) AAAD involving arch vessels, and (2) an intimal tear located in the transverse arch or proximal descending aorta that could not be resected by hemiarch replacement alone. Patients with mitral and tricuspid valve lesion and coronary heart disease requiring surgery were excluded.

This study was approved by the ethics committee of Union Hospital, Fujian Medical University and conformed to the Declaration of Helsinki.

### Modified triple-branched stent graft

**Fig. 1:**
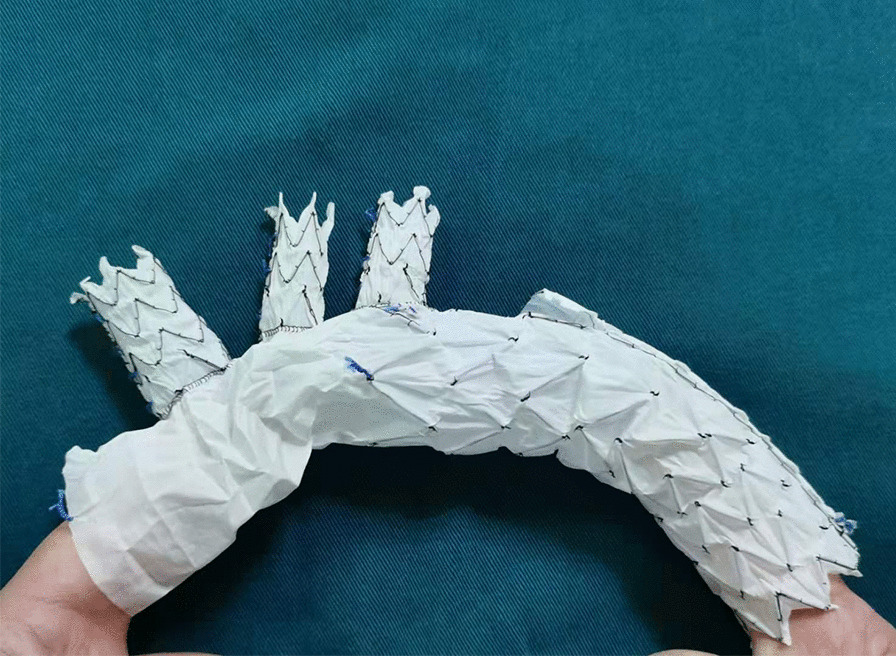
The modified triple-branched stent graft is comprised of a main graft and three sidearm grafts

The modified triple-branched stent graft used in this study was conceived and designed by Dr. Chen [5–7]. It consists of a self-expandable nitinol stent and a Dacron vascular graft (Yuhengia Sci Tech Corp Ltd, Beijing, China; Fig. 1). The Dacron graft is comprised of a main graft and three sidearm grafts, which are commercialized in China as individual devices. We assembled the individual pieces into the triple-branched stent graft during the operation [5–7].

### Surgical technique

**Fig. 2:**
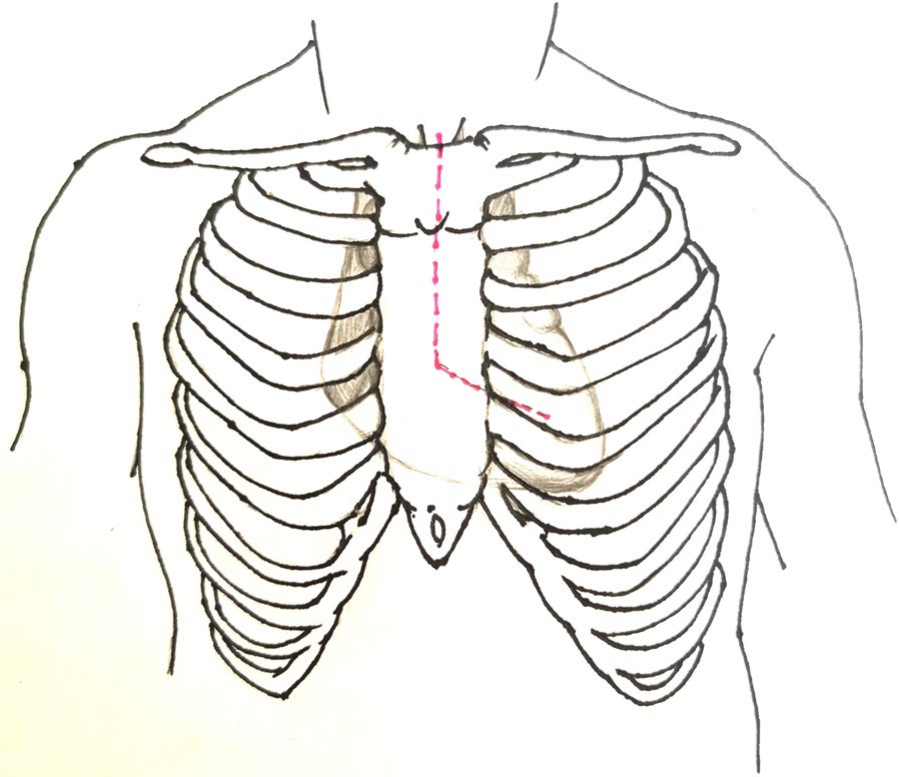
Partial upper sternotomy incision for extensive arch repair of acute type A aortic dissection

**Fig. 3:**
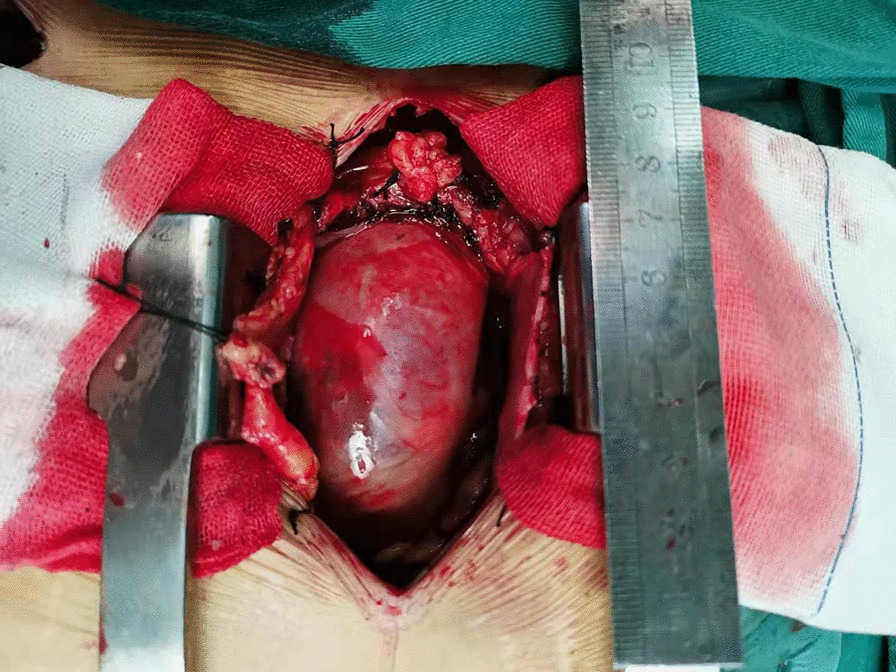
Excellent exposure of acute type A aortic dissection is achieved through a partial upper sternotomy approach

Patients were placed in the supine position under general anesthesia. The PUS (“L” shape) was constructed between the sternal notch and the level of fourth intercostal space, and then extended to the left fourth intercostal space (Fig. 2). The initial skin incision was approximately 10–15 cm, but it was extended when surgical exposure was insufficient. After opening the pericardium, we fixed the pericardium to drapes with several stay sutures, which elevated the heart and the aortic arch anteriorly and offered excellent exposure (Fig. 3).

Details of the extensive arch repair procedure have been previously described [5–7]. Cardiopulmonary bypass is first established by cannulation of the right axillary and femoral arteries and direct right atrial cannulation. When the nasopharyngeal temperature reached 32 °C, an aortic cross-clamp was applied, and aortic root reconstruction was performed. The reconstructed aortic root was anastomosed to a 3–4 cm straight Dacron tube graft (Boston Scientific, Inc, Natick, MA) under moderate hypothermic circulatory lower body arrest (25 °C) with selective antegrade cerebral perfusion via the right axillary cannula. After resection of the lesser curvature, the brachiocephalic vessel orifices and the true lumen of descending aorta were exposed, and the modified triple-branched stent graft were deployed one by one into the true lumen of the brachiocephalic vessel, arch, and descending aorta. The distal Dacron tube graft was directly anastomosed to the reserved arch stump, thus incorporating the proximal end of the triple-branched stent graft with a continuous suture.

### Follow up

All survivors were followed up through phone calls or e-mails. CTA of thoracic and abdominal aorta and echocardiography were performed before discharge, 3 months and 6 months after surgery, and annually thereafter.

### Statistical analysis

All analyses were performed using SPSS 25.0 and R 3.6.3. Univariate analyses were performed with the Chi-square test for categorical variables, and Student-t test or Wilcoxon-Mann–Whitney test for continuous variables. Logistic regression analyses were used to study the association between the potential risk factors for AAAD and early death. Negative binominal regression was used to estimate the relation between the post-operative ventilator-supporting time (hours) or intensive care unit (ICU) stay time (days) and potential risk factors. Log-rank was used to calculate the P-value that corresponds with differences in the 2-year survival rate.

## Results

### Patient characteristics

**Fig. 4:**
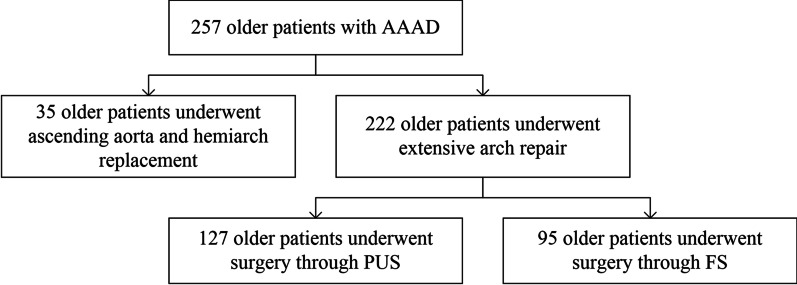
There were 257 patients with acute type A aortic dissection who underwent the surgical procedure between January 2014 and December 2019

Between January 2014 and December 2019, 257 older adult patients with AAAD were admitted to our hospital. Among them, 222 patients underwent extensive arch repair (127 PUS, 95 FS), 35 patients were excluded from undergoing extensive arch repair and instead underwent ascending aorta and hemiarch replacement. A Consolidated Standards of Reporting Trials (CONSORT) diagram is shown in Fig. 4.

Tables 1 and 2 present preoperative and operative, and postoperative patent data, respectively. The PUS group had a shorter Cardiopulmonary bypass time (133.0 vs 155.0 min, *P* < 0.001), cross-clamp time (44.0 vs 61.0 min, *P* < 0.001) and selective cerebral perfusion time (11.0 vs 21.0 min, *P* < 0.001) than the FS group. The PUS group had a less mediastinal drainage (350.0 vs 500.0 ml, < 0.001) and red blood cell transfusion (4.0 vs 6.0 unit, < 0.001). There were no significant differences in any other preoperative and operative variables.

**Table 1:** Preoperative characteristics

Characteristics	Overall (n = 222)	Full sternotomy (n = 95)	Partial upper sternotomy (n = 127)	*P* value
Age (y)	63.0 (61.0, 67.0)	62.0 (60.0, 67.0)	61.0 (63.0, 67.0)	0.216
Male (n, %)	156 (70.3)	73 (76.8)	83 (65.4)	0.064
Marfan syndrome (n, %)	2 (0.9)	1 (1.1)	1 (0.8)	0.837
Hypertension (n, %)	190 (85.6)	80 (84.2)	110 (86.6)	0.614
Diabetes (n, %)	20 (9.0)	10 (10.5)	10 (7.9)	0.495
Serum Creatinine (umol/L)	95.0 (76.0, 123.5)	98.0 (81.0, 121.0)	90.0 (74.0, 127.0)	0.624
Body mass idext(BMI) (kg/m^2^)	25.1 ± 1.4	25.1 ± 1.0	25.2 ± 1.6	0.880
Left ventricle ejection fraction (LVEF) (%)	64.3 (60.1, 67.2)	64.4 (60.1, 67.8)	64.1 (60.1, 66.9)	0.545
Aortic regurgitation (AR) ≧moderate (n, %)	42 (18.9)	14 (14.7)	28 (22.0)	0.169
Pericardial effusion (n, %)	6 (2.7)	1 (1.1)	5 (3.9)	0.372
Organ malperfusion (n, %)				
Heart	5 (2.3)	2 (2.1)	3 (2.4)	0.999
Cerebral	5 (2.3)	3 (3.2)	2 (1.6)	0.742
Renal	26 (11.7)	10 (10.5)	16 (12.6)	0.635
Iliofemoral	9 (4.1)	4 (4.2)	5 (3.9)	0.999

Continuous variables were shown as mean (SD) or median (Q25,Q75); Categorical variables were shown as number (%). The Student t test or Man-Whitney U test was used for continuous variables, and Chi-square test used for categorical variables

**Table 2:** Operative and postoperative data

Variables	Overall (n = 222)	Full sternotomy (n = 95)	Partial upper sternotomy (n = 127)	*P* value
**Operative data**				
Cardiopulmonary bypass (min)	141.0 (125.0, 168.0)	155.0 (133.0, 180.0)	133.0 (120.0, 160.0)	< 0.001
Cross-clamp time (min)	51.0 (39.0, 65.3)	61.0 (50.0, 68.0)	44.0 (36.0, 58.0)	< 0.001
Selective cerebral perfusion time (min)	15.0 (11.0, 21.0)	21.0 (18.0, 25.0)	11.0 (10.0, 14.0)	< 0.001
**Aortic root procedure**				
Bentall (n, %)	18 (8.1)	8 (8.4)	10 (7.8)	0.307
Aortic valve repalcement (n, %)	4 (1.8)	0 (0.0)	4 (3.1)	0.217
Reconstruction of sinus of Valsava (n, %)	110 (49.5)	45 (47.4)	65 (51.2)	0.271
**Postoperative data**				
Mediastinal drainage (ml)	400.0 (300.0, 585.0)	500.0 (370.0, 700.0)	350.0 (300.0, 450.0)	< 0.001
Red blood cell transfusion(unit)	4.0 (4.0, 6.0)	6.0 (4.0, 8.0)	4.0 (3.0, 6.0)	< 0.001
Ventilator-supporting time (hours)	57.0 (35.8, 80.3)	57.0 (35.0, 96.0)	57.0 (36.0, 76.0)	0.469
ICU stay time (days)	5.0 (3.0, 9.0)	5.0 (3.0, 7.0)	6.0 (4.0, 9.0)	0.184
Re-do for bleeding (n, %)	3 (1.4)	3 (3.2)	0 (0.0)	0.153
Neurologic dysfunction (n, %)	15 (6.8)	6 (6.3)	9 (7.1)	0.821
Acute kidney injury (n, %)	40 (18.0)	16 (16.8)	24 (18.9)	0.693
Hepatic insufficiency (n, %)	61 (27.5)	33 (34.7)	28 (22.0)	0.036
Gastrointestinal hemorrhage (n, %)	32 (14.4)	20 (21.1)	12 (9.4)	0.015
Tracheotomy (n, %)	10 (4.5)	4 (4.2)	6 (4.7)	0.999
Early death (n, %)	18 (8.1)	9 (9.5)	9 (7.1)	0.519

Continuous variables were shown as mean (SD) or median (Q25,Q75); Categorical variables were shown as number (%). The Student t test or Man-Whitney U test was used for continuous variables, and Chi-square test used for categorical variables

### Early outcomes

The total 30-day mortality was 8.1% (18/222). In postoperative complications, The PUS group had a less hepatic insufficiency (22.0% vs 34.7%, *p* = 0.036) and gastrointestinal hemorrhage (9.4% vs 21.1%, *p* = 0.015) (Table 2).

Table 3 presents the univariate and multivariate analysis findings for 30-day mortality. Multivariate analysis demonstrated that LVEF < 50% (OR 17.05; 95% CI 1.87–155.63; *P* = 0.012) and malperfusion syndromes (OR 65.83; 95% CI 11.53–375.86; *P* < 0.001) were related to early death.

**Table 3:** Negative binomial regression analysis of potential risk factors for post-operative ventilator-supporting time (hours)

Valuables	Univariate		Multivariate ^a^	
N = 204	*P*	IRR (95%CI)	*P*	IRR (95%CI)
Male gender	0.140	1.17 (0.95, 1.44)	–	–
Age	0.806	1.00 (0.98, 1.02)	–	–
Mafan syndrome	0.973	1.02 (0.39, 2.68)	–	–
Hypertension	< 0.001	1.73 (1.34, 2.23)	0.001	1.59 (1.25, 2.03)
Diabetes	0.009	1.55 (1.11, 2.16)	0.020	1.43 (1.06, 1.93)
Elevated creatinine	0.014	1.32 (1.06, 1.64)	0.272	1.13 (0.91, 1.41)
BMI ≥ 24	0.816	0.97 (0.77, 1.23)	–	–
LVEF < 50%	0.026	1.80 (1.07, 3.00)	0.027	1.69 (1.06, 2.07)
AR	0.270	1.15 (0.90, 1.46)	–	–
Malperfusion syndromes	0.040	1.38 (1.01, 1.88)	0.046	1.36 (1.00, 1.83)
Partial upper sternotomy	< 0.001	0.71 (0.59, 0.86)	0.003	0.76 (0.64, 0.91)
Cardiopulmonary bypass time^b^	0.001	1.10 (1.04, 1.17)	0.006	1.07 (1.02, 1.13)

^a^The variables with *P* < 0.05 in univariate analysis were further involved in the multivariate analysis. ^b^Cardiopulmonary bypass time was grouped by each 20 minutes

Table 4 present the negative binomial regression analysis of potential risk factors for post-operative ventilator-supporting time and post-operative ICU stay time. In a multivariate model, the PUS approach (IRR 0.76; 95% CI 0.64–0.91; *P* = 0.003) contributed to shorter ventilator-supporting time, but hypertension (IRR 1.59; 95% CI 1.25–2.03; *P* = 0.001), diabetes (IRR 1.43; 95% CI 1.06–1.93; *P* = 0.020), LVEF < 50% (IRR 1.69; 95% CI 1.06–2.07; *P* = 0.027), malperfusion syndromes (IRR 1.36; 95% CI 1.00–1.83; *P* = 0.046), cardiopulmonary bypass time (IRR 1.07; 95% CI 1.02–1.13; *P* = 0.006) leaded to longer ventilator-supporting time. Hypertension (IRR 1.39; 95% CI 1.05–1.83; *P* = 0.020), elevated creatinine (IRR 1.31; 95% CI 1.04–1.66; *P* = 0.024), malperfusion syndromes (IRR 1.37; 95% CI 1.00–1.86; *P* = 0.047), cardiopulmonary bypass time (IRR 1.06; 95% CI 1.01–1.12; *P* = 0.019) leaded to longer post-operative ICU stay time.

**Table 4:** Negative binomial regression analysis of potential risk factors for post-operative ICU stay time (days)

Valuables	Univariate		Multivariate ^a^	
N = 204	*P*	IRR (95%CI)	*P*	IRR (95%CI)
Male gender	0.195	1.16 (0.93, 1.45)	–	–
Age	0.259	1.01 (0.99, 1.04)	–	–
Mafan syndrome	0.962	0.98 (0.35, 2.74)	–	–
Hypertension	0.007	1.48 (1.11, 1.98)	0.020	1.39 (1.05, 1.83)
Diabetes	0.044	1.42 (1.01, 2.00)	0.106	1.31 (0.94, 1.83)
Elevated creatinine	< 0.001	1.52 (1.22, 1.91)	0.024	1.31 (1.04, 1.66)
BMI ≥ 24	0.530	0.92 (0.72, 1.18)	**–**	**–**
LVEF < 50%	0.039	1.73 (1.03, 2.93)	0.053	1.63 (0.99, 2.68)
AR	0.797	1.04 (0.80, 1.34)	**–**	**–**
Malperfusion syndromes	0.015	1.47 (1.08, 2.01)	0.047	1.37 (1.00, 1.86)
Partial upper sternotomy	0.793	0.97 (0.79, 1.19)	**–**	**–**
Cardiopulmonary bypass time	0.021	1.07 (1.01, 1.13)	0.019	1.06 (1.01, 1.12)

^a^The variables with *P* < 0.05 in univariate analysis were further involved in the multivariate analysis

### Follow up

**Fig. 5:**
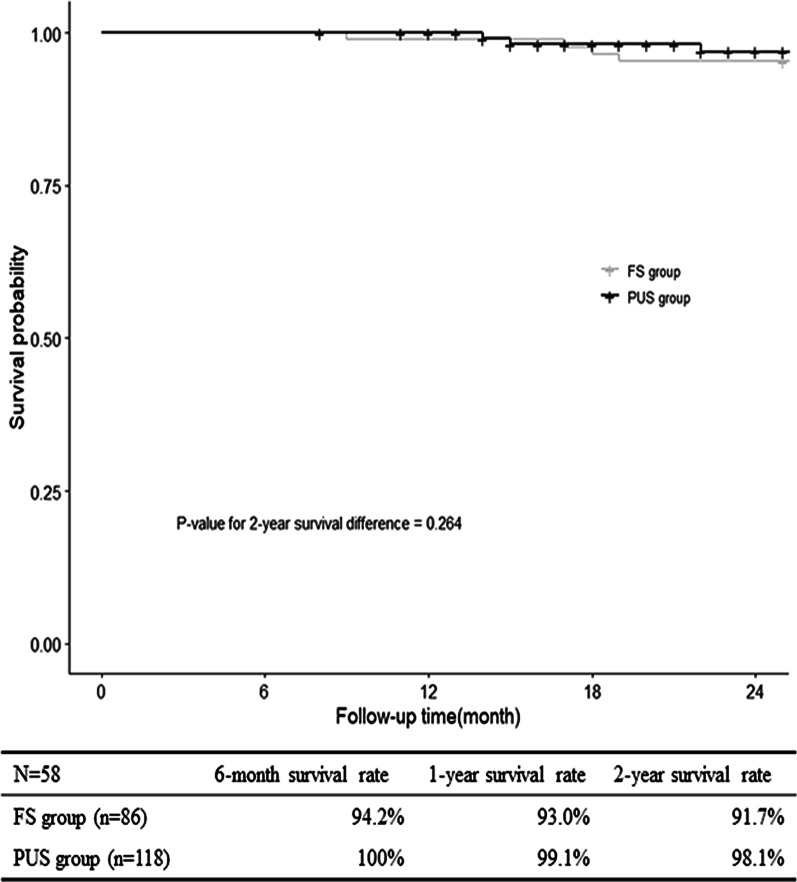
Kaplan–Meier estimates of survival for patients with acute type A aortic dissection who underwent extensive arch repair with modified triple-branched stent graft

There were 204 patients who survived the procedure and were followed up. The mean follow-up duration was 44.3 ± 24.8 months. The actuarial survival rate at 6 months, 1 year, 2 years was 100%, 99.1%, 98.1% in the PUS group, respectively; and was 94.2%, 93.0%, 91.7% in FS group, respectively. There was no significant difference in survival rate between the two groups (Fig. 5).

## Discussion

With increased life span, cardiovascular disease, including AAAD, has seriously harmed the overall health of older adults. Older age is generally considered as a risk factor for surgical intervention for AAAD. The hospital mortality due to surgical repair for AAAD in older adults is high in some reports [8, 9]. Despite advances in surgical techniques, it continues to be a frustrating challenge for cardiac surgeons.

Reports of aortic valve replacement, even aortic arch surgery, utilizing a PUS approach have been published [10–14]. However, there are few reports regarding the use of a PUS during AAAD in older adults. Although the association between surgical volume and outcomes for AAAD have not been demonstrated, our group has experienced more than 1000 open surgeries for modified triple-branched stent graft in AAAD since 2014, and we have established an efficient process of diagnosis and treatment of AAAD. Based on these studies, since 2017 we have adopted the PUS approach for extensive arch repair for AAAD in older adults. The PUS approach has proven to be a feasible and safe approach for aortic valve replacement and aortic root surgery. For example, Inoue and colleagues published initial results of 15 cases of total arch replacement utilizing PUS. In their procedure, they used both an “L” shape and “T” shape for PUS [13]. The “L” shape utilized in our study provided excellent exposure, with no conversion to FS required. Interestingly, among our patients, the PUS group presented shorter Cardiopulmonary bypass time, cross-clamp time and shorter selective cerebral perfusion time than the FS group. It is possible surgical skill of the performing surgeon may improve with the increase in surgical volume.

In our cohort, the results are similar to those reported in the literature, ranging from 13.3% to 45.6% in older adults patients with AAAD in previous studies [4, 8, 9, 15–17]. Our study demonstrated that LVEF < 50% and malperfusion syndromes were significant risk factors of early death in older adult surgical patients with AAAD [18–21]. Dissection-related factors could lead to a progressive worsening of hemodynamic instability and organ function. Likely, these patients were in left ventricular dysfunction or multiple organ failure after surgery, eventually leading to death. The treatment strategy for these patients was to establish cardiopulmonary bypass as soon as possible, securing true lumen flow and restoring organ perfusion.

Prolonged operation or cardiopulmonary bypass time may be harmful for older adult patients. Several research institutions recommend surgery with only ascending aorta replacement for older adult patients with AAAD [8, 22–24]. However, our novel technique could simplify extensive arch repair, reducing surgery time. Our results demonstrated that cardiopulmonary bypass time, cross-clamp time, selective cerebral perfusion time were 133.0 min, 44.0 min, and 11.0 min in the PUS group. Previous studies have reported these variables ranged from 214 to 223 min, 125 to 146 min, and 54 to 69 min, respectively [14, 25]. It is possible that our novel technique and experience of a large surgical volume of AAAD lead to shorter surgical times.

Several studies have demonstrated that compared with a full sternotomy, PUS provided earlier extubation, less blood loss, less blood transfusion, and less incisional pain [12, 26]. In our study, the PUS group had a less mediastinal drainage and red blood cell transfusion. In the multivariate model, the PUS approach contributed to shorter ventilator-supporting time compared with the FS group. The PUS approach reduced surgical trauma, and maintained chest stability, which contributed to the recovery of postoperative respiratory function. Furthermore, PUS offers a rapid postoperative recovery and cosmetic advantages.

The hypertension, malperfusion syndromes and cardiopulmonary bypass time both leaded to longer ventilator-supporting time and post-operative ICU stay time. The hypertension may be the most common predisposing factor for AAAD. A sharp rise in blood pressure and tearing of aorta could lead to a systemic inflammatory response. And during long CPB time, the exposure of blood to abnormal surfaces may induce a systemic inflammatory response. All these factors could contribute to acute respiratory distress syndrome, which prolonged ventilator-supporting time and post-operative ICU stay time.

The significant limitation of the study is that it is a retrospective study, and has a lack of statistical power due to small sample sizes. A prospective randomized controlled trial is required to evaluate this result.

## Conclusion

Our study is report extensive arch repair for older adult patients with AAAD through PUS, which obtained satisfactory early results. However, the long-term results of the PUS approach requires further follow-up.

## Data Availability

The data of this study will not be shared publically because they will be applied for further researches of this series. But corresponding authors do agree that the data can be shared individually if requested.

## Abbreviations

AAAD: Acute type A aortic dissection
FS: Full sternotomy
PUS: Partial upper sternotomy
CTA: Contrast-enhanced computed tomography angiography
OR: Odds ratio
IRR: Incidence rate ratio
CI: Confidence interval
AR: Aortic regurgitation
BMI: Body mass index
LVEF: Left ventricle ejection fraction
